# Supplementary material for: Synthesis, Characterization, and DFT Calculations of a New Sulfamethoxazole Schiff Base and Its Metal Complexes
Source: Materials (Basel). 2023 Jul 21;16(14):5160. doi: 10.3390/ma16145160 (PMC10385116; doi:10.3390/ma16145160)
Supplement: Supplementary file 1 [file materials-16-05160-s001.zip › materials-2462250-supplementary.pdf]

Supplementary Information

Synthesis, Characterization, and DFT Calculations of a New Sulfamethoxazole Schiff Base and Its Metal Complexes

Jibril I. Al-Hawarin [1], Abdel-Aziz Abu-Yamin [1,*], Abd Al-Aziz A. Abu-Saleh [2], Ibrahim A. M. Saraireh [1], Mansour H. Almatarneh [2,3], Mahmood Hasan [4], Omar M. Atrooz [5] and Y. Al-Douri [6,7,8,*]

[1] Department of Chemistry, Al-Hussein Bin Talal University, Ma'an 71111, Jordan
[2] Department of Chemistry, Memorial University, St. John's, NL A1B 3X7, Canada
[3] Department of Chemistry, University of Jordan, Amman 11942, Jordan
[4] Hepi Company (Home of Experience) for Paints and Inks, Cairo 61710, Egypt
[5] Department of Biological Sciences, Mutah University, Mutah 617102, Jordan
[6] Nanotechnology and Catalysis Research Center (NANOCAT), University of Malaya, Kuala Lumpur 50603, Malaysia
[7] Department of Mechanical Engineering, Faculty of Engineering, Piri Reis University, Eflatun Sk. No: 8, Istanbul 34940, Tuzla, Turkey
[8] Department of Applied Physics and Astronomy, College of Sciences, University of Sharjah, Sharjah P.O. Box 27272, United Arab Emirates
* Correspondence: abuyamin@ahu.edu.jo (A.-A.A.-Y.); yaldouri@yahoo.com (Y.A.-D.)

Table of contents:

Table S1. Cartesian coordinates (Å) of the optimized structure of the Schiff base at the M06-2X/6-31G(d) level of theory.

Table S2. Calculated principal (most intense) excitation energies of the Schiff base

Figure S1: FTIR spectra of L, L1, L2, L3, and L4.

Figure S2: ^{1}H and ^{13}C- NMR spectra of Schiff base.

Figure S3: MS spectrum of Schiff base.

Table S1. Cartesian coordinates (Å) of the optimized structure of the Schiff base at the M06-2X/6-31G(d) level of theory.

C	4.31789700	2.73908800	-0.07805000

C	4.53959500	1.47015300	-0.50799000
C	4.23961500	0.69277000	0.64833600
H	4.87394100	1.11813600	-1.47027200
C	4.43022600	4.07410800	-0.72426100
H	4.76626500	3.96122800	-1.75570100
H	3.46361200	4.58554400	-0.71984800
H	5.14597500	4.70046400	-0.18486000
O	3.93094300	2.72596500	1.20835700
N	3.86656100	1.42796900	1.66372400
N	4.34995700	-0.69340500	0.80621800
H	4.16473900	-1.01066300	1.75599700
S	3.66181300	-1.71886000	-0.34100600
O	3.88126200	-3.04924600	0.19080000
O	4.18093000	-1.28554200	-1.62421100
C	1.92211500	-1.38065800	-0.31052500
C	1.41266300	-0.33980000	-1.08789000
C	1.09860800	-2.14736300	0.50888800
C	0.05685600	-0.05976900	-1.02993600
H	2.07311600	0.21897400	-1.74295500
C	-0.25716600	-1.85375600	0.56866300
H	1.51847400	-2.97794600	1.06744800
C	-0.78600500	-0.79373800	-0.18304700
H	-0.37815000	0.73072300	-1.63175900
H	-0.92149400	-2.46776800	1.16858100
N	-2.14732500	-0.46550700	-0.17224900
C	-2.77088700	-0.46093200	0.94469700
H	-2.24331100	-0.65150000	1.88925800
C	-4.20087200	-0.18764200	1.04367200
H	-4.62980400	-0.17497600	2.04207300
C	-4.96652500	0.04042700	-0.03909400
H	-4.50075800	0.01941300	-1.02191200
C	-6.37964000	0.31744900	-0.00842100
O	-6.97835100	0.51715400	-1.21696100
C	-7.31576500	0.43471700	0.98560500
C	-8.28342400	0.75873900	-0.98895500
C	-8.55427800	0.72191700	0.34479800
H	-7.13703400	0.32870400	2.04576400
H	-8.89001700	0.93732400	-1.86315600
H	-9.51525100	0.87980700	0.81076300

Table S2. Calculated principal (most intense) excitation energies of the Schiff base

Excited state	Energy (eV)	λ (nm)	Oscillator strength
1st excited state (HOMO → LUMO)	3.516	352	1.251
2nd excited state	4.092	303	0.029
3rd excited state	5.031	247	0.002

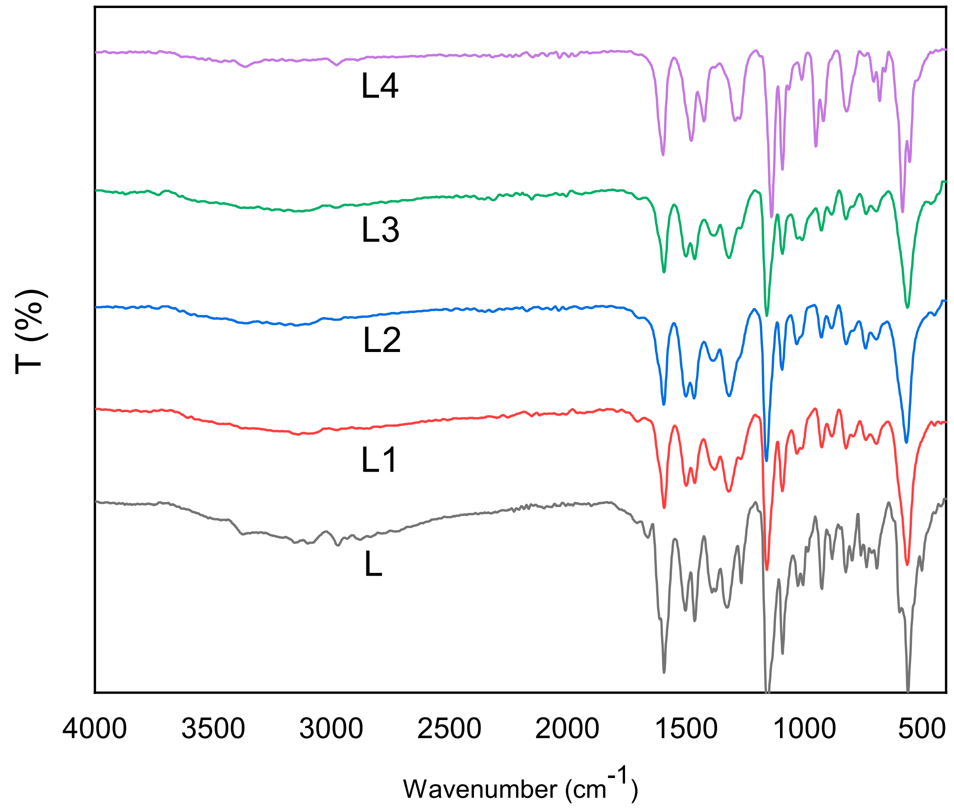

Figure S1: FTIR spectra of L, L1, L2, L3, and L4.

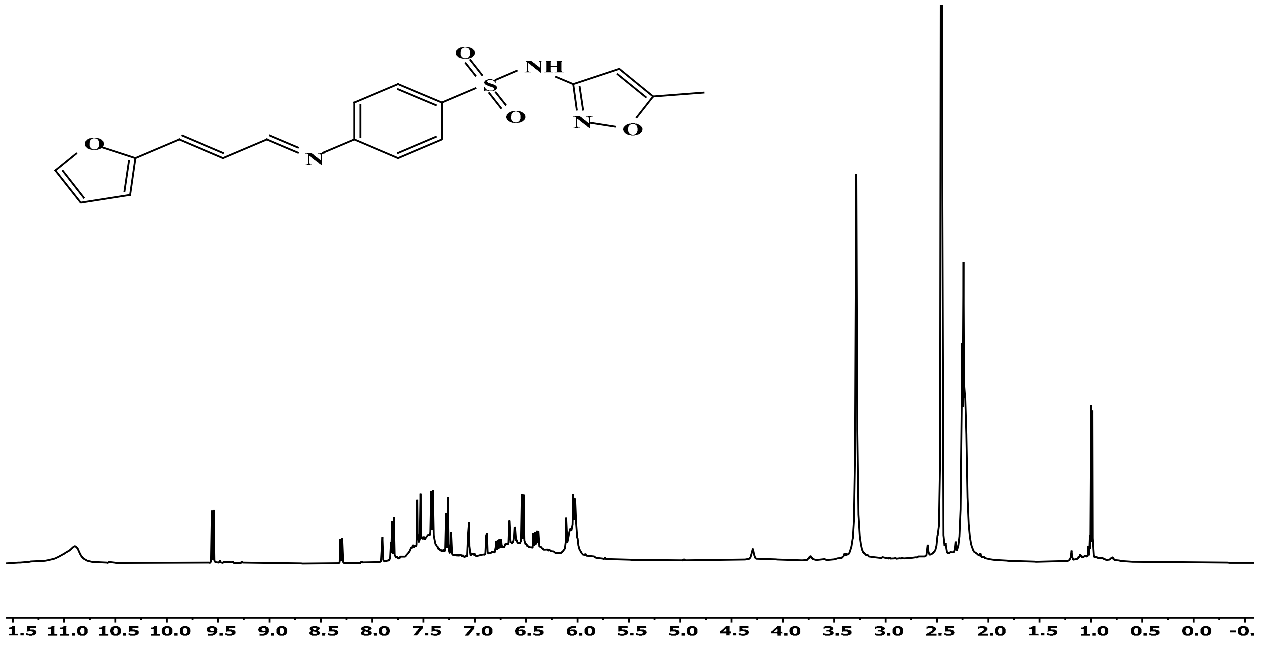

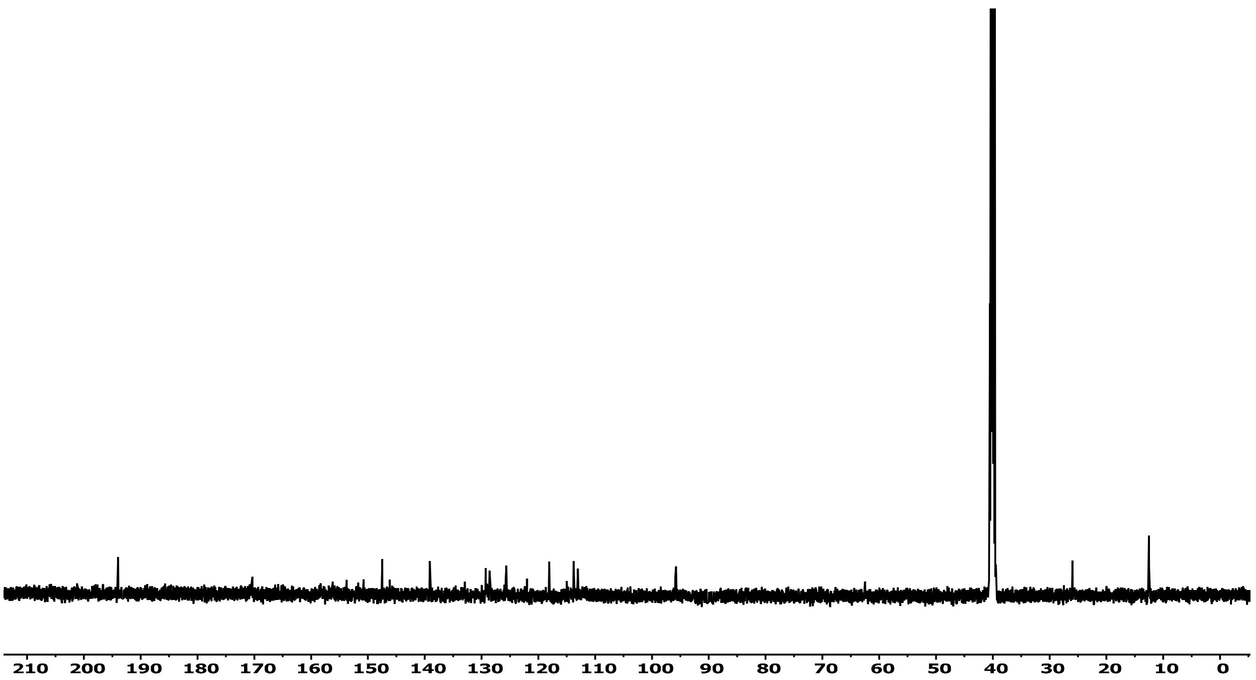

Figure S2: ^{1}H and ^{13}C- NMR spectra of Schiff base.

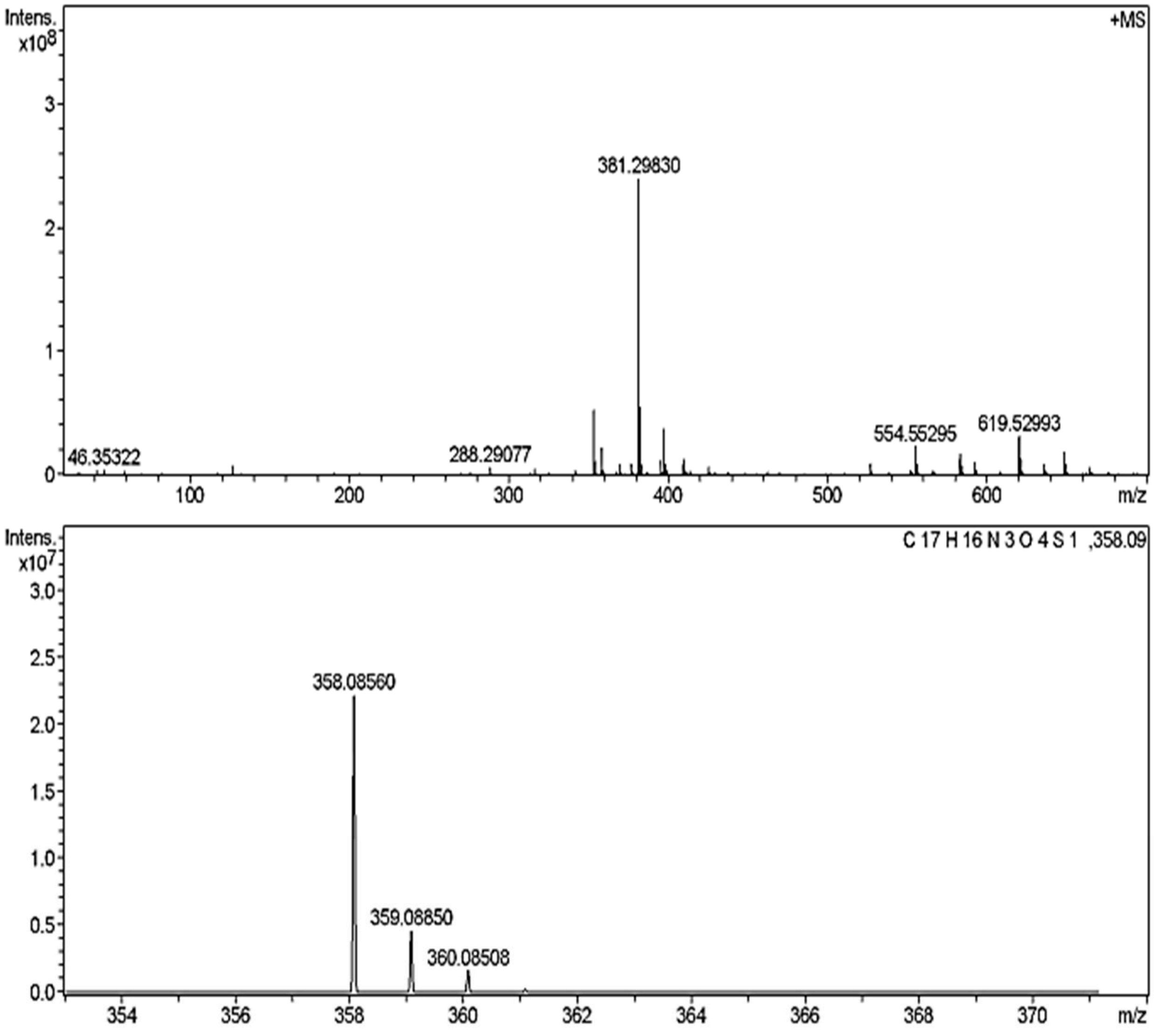

Sum Formula	Sigma	m/z	Err [ppm]	Mean Err [ppm]	Err [mDa]	rdb	N Rule	e^-
C 17 H 16 N 3 O 4 S 1	0.013	358.08560	-3.32	-3.15	-1.19	11.50	ok	even

Figure S3: MS spectrum of Schiff base.
